# ERK-mediated Cytoplasmic Retention of USP11 Contributes to Breast Cancer Cell Proliferation by Stabilizing Cytoplasmic p21

**DOI:** 10.7150/ijbs.71327

**Published:** 2022-03-21

**Authors:** Ling Li, Tanggang Deng, Lin Zhang, Yanpeng Wang, Yangyang Zhou, Yan Liu, Hui Li, Jing Dai, Yuan Yang, Neng Ling, Huimin Liu, Jing Liu, Lanqin Cao, Min Xu, Mao Ye

**Affiliations:** 1 Molecular Science and Biomedicine Laboratory (MBL), State Key Laboratory of Chemo/Biosensing and Chemometrics, College of Biology, College of Chemistry and Chemical Engineering, Aptamer Engineering Center of Hunan Province, Hunan University, Changsha, Hunan 410082, China.; 2 Molecular Biology Research Center and Center for Medical Genetics, School of Life Sciences, Central South University, Changsha, Hunan 410078, China.; 3 Department of Gynecology, Xiangya Hospital, Central South University, Changsha, Hunan 410078, China.; 4 Department of Critical Care Medicine, the Second Xiangya Hospital, Central South University, Changsha, Hunan 410011, China.

**Keywords:** Cytoplasmic p21, ERK1/2, Phosphorylation, Stabilization, USP11

## Abstract

Breast cancer ranks as the most frequently diagnosed cancer among women worldwide. Elevated cytoplasmic p21 levels are often found in breast cancer tissues and related to a poor prognosis. However, the underlying mechanisms that lead to the stabilization of cytoplasmic p21 protein, which normally has a very short half-life, remain obscure. In this study, we found that there was a strong correlation between p21 and USP11 in the cytoplasm of breast cancer tissues and cells. Furthermore, we revealed that ERK1/2 phosphorylated USP11 at the Ser905 site, which promoted the cytoplasmic localization of USP11. In the cytoplasm, USP11 colocalized and interacted with p21. As a result, USP11 catalyzed the removal of polyubiquitin chains bound to cytoplasmic p21 and resulted in its stabilization. Functionally, USP11-mediated stabilization of cytoplasmic p21 induced breast cancer cell proliferation* in vitro* and *in vivo*. Our findings provide the first evidence that ubiquitinated p21 in the cytoplasm can be recycled through USP11-mediated deubiquitination, and we identified the USP11-p21 axis in the cytoplasm as a potential therapeutic target for breast cancer control.

## Introduction

Breast cancer was the most common cancer diagnosed among women worldwide in 2020, with an estimated 2.3 million new cases 1. Despite the great progress in diagnostic and therapeutic approaches, such as surgery, chemotherapy, radiotherapy, endocrine therapy, and immunotherapy, breast cancer is still the leading cause of cancer death in women (15.5% of the total cancer deaths) 1-6. Therefore, there is an immediate need to unravel the molecular mechanisms in breast cancer tumorigenesis and progression for effectively controlling breast cancer.

p21 is encoded by the *CDKN1A* gene, which can function as an oncogenic protein or a tumor suppressor, mainly depending on its subcellular localization 7-9. Nuclear p21 acts as a tumor suppressor that arrests the cell cycle at G1 and G2 phases by suppressing cyclin/CDK complexes activity and PCNA-dependent DNA replication 10, 11. In contrast, cytoplasmic p21 is considered to be an oncoprotein that enhances tumor cell proliferation 12, decreases apoptosis 7, 13-17, increases chemoresistance 18-23, and induces migration and invasion 24. Elevated cytoplasmic p21 levels are often found in breast cancer tissues 25, and its overexpression predicts poor outcomes in breast cancer patients 26. Increasing evidence indicates that cytoplasmic p21 promotes breast cancer cell proliferation, migration and invasion 27, 28.

p21 protein levels are mainly regulated by two posttranslational modifications, namely phosphorylation and ubiquitination 29. Phosphorylation events mainly impact the subcellular localization of p21. For instance, ERK2-mediated phosphorylation leads to cytoplasmic localization of p21 30. Hyperactivation of the ERK pathway often contributes to breast cancer initiation and progression 31. Moreover, ubiquitination is primarily involved in the control of p21 protein levels 32, 33. In the nucleus, three E3 ubiquitin ligase complexes, APC/C^CDC20^, CRL4^CDT2^, and SCF^SKP2^, have been shown to promote ubiquitination and degradation of p21. Our previous study further demonstrated that USP11 could reverse the nuclear p21 degradation mediated by SCK^SKP2^, APC/C^CDC20^ and CRL4^CDT2^ by removing the polyubiquitin chains bound to p21, and stabilize p21 34. In the cytoplasm, the E3 ubiquitin ligase complex CRL2^LRR1^ has been revealed to promote p21 degradation via ubiquitination 35. However, it remains unknown whether cytoplasmic p21 can be recycled.

USP11 belongs to the ubiquitin-specific processing protease family of deubiquitinases, which regulates DNA damage repair, proliferation and metastasis in multiple cancer types by specifically interacting with and deubiquitinating target proteins 36, 37. Previous studies have suggested that USP11 functions as a tumor suppressor and oncogenic protein. The tumor-suppressive activities of USP11 were found in non-small cell lung cancer 34, brain tumors 38 and squamous cell carcinoma 39. In contrast, USP11 plays a tumor-promoting role in hepatocellular carcinoma 40, melanoma 41, gastric cancer 42, and breast cancer 43. However, the underlying mechanism that USP11 has contradictory effects on tumor development in different types of tumors remains obscure.

The biofunction of a protein is always associated with its subcellular localization 44. Phosphorylation modifications play a significant role in the subcellular localization of deubiquitinases. For example, the subcellular localization of USP10 is regulated by ATM-mediated phosphorylation of USP10 45. It is poorly known whether there exists a phosphorylation modification to regulate subcellular localization of USP11.

In this study, we found that there was a strong correlation between p21 and USP11 in the cytoplasm of breast cancer tissues and cells. We provided evidence that ubiquitinated p21 in the cytoplasm could be reversed and stabilized by USP11-mediated deubiquitination. We also demonstrated that the cytoplasmic localization of USP11 was associated with its phosphorylation mediated by ERK1/2. As a result, USP11-mediated stabilization of cytoplasmic p21 promoted the proliferation of breast cancer cells. Our results reveal an important mechanism regarding the regulation of cytoplasmic p21 stability, and indicate that the USP11-p21 axis in the cytoplasm could be a potential therapeutic target for breast cancer control.

## Materials and Methods

### Bioinformatics analysis

We conducted USP11 and p21 protein expression analysis using the UALCAN portal 46.

### Plasmid transfection, RNA interference and Lentivirus infection

Plasmids were transfected into cells using Lipomax (SUDGEN, 32011). The sequences of the small interfering RNAs (siRNAs) used in this study were shown in Supplementary Table S1. siRNAs were transfected into cells using GenMute^TM^ siRNA Transfection Reagent. To stably knock down endogenous USP11 in MCF-7 cells, cells were infected with negative control (NC) lentivirus or USP11-shRNA lentivirus (purchased from GenePharma) for 72 h and subsequently selected with 2 μg/mL puromycin for 5 days. The shRNA target sequences were shown in Supplementary Table S2.

### Reagents and antibodies

U0126 (catalog no. S1901) and EGF (catalog no. P5552) were purchased from Beyotime Biotechnology. Mitoxantrone (catalog no. HY-13502) was purchased from MCE. TureColor three-color pre-stained protein Marker (catalog no. C510010) was purchased from Sangon Biotech. BSA (catalog no. B7004M) was purchased from US EVERBRIGHT. UltraSignal ECL (catalog no. 4AW011-100) was purchased from 4A Biotech Co., Ltd. Antibodies information: anti-USP11 (Santa Cruz, catalog no. sc-365528/ Abcam, catalog no. ab109232); anti-p21 (Santa Cruz, catalog no. sc-397/ Cell Signaling Technology, catalog no. 2947S); anti-Flag (MBL, catalog no. M185-3L); anti-Myc (MBL, catalog no. M192-3); anti-HA (MBL, catalog no. M180-3); anti-ERK1/2 (Cell Signaling Technology, catalog no. 4695S); anti-phospho-ERK1/2 (Cell Signaling Technology, catalog no. 4376S); anti-phospho-MAPK/CDK Substrates (Cell Signaling Technology, catalog no. 2325S); anti-phosphoserine/threonine (BD Transduction Laboratories, catalog no. M180-3); anti-GAPDH (COOLRUN Life Science, catalog no. AT0002); Dylight 488 (Thermo Fisher Scientific, catalog no. #35502); Dylight 594 (Thermo Fisher Scientific, catalog no. #35560).

### Cell culture

MDA-MB-468, HEK293, MCF-7 and 4T1 cells were purchased from ATCC. SKBR3 and BT474 was obtained from Jining Corporation. MCF-7, MDA-MB-468, HEK293, and 4T1 cells were cultured in DMEM containing 10% FBS and 1% Pen/Strep. BT474 and SKBR3 cells were cultured in RPMI-1640 containing 10% FBS and 1% Pen/Strep. All cells were maintained at 37 °C with 5% CO2. Cells were stored at -80 °C using CELLSAVING (New Cell & Molecular Biotech).

### Quantitative Real‑time PCR

Total RNA was acquired using RNA Extraction Reagents (Beyotime Biotechnology, catalog no. R0026). Then total RNA was subjected to reverse transcription using Nova UScript First-Stand cDNA Synthesis SuperMix (Innovagene catalog no. AR111). qRT-PCR reaction consisted of the resulting cDNA, primer, and 2× SYBR Green qPCR Master Mix (Bimake, catalog no. B21702). The primer sequences for qRT-PCR were shown in Supplementary Table S3.

### Immunofluorescence Staining

Cells were seeded in confocal dishes for 1 day, then washed once with DPBS. The cells were fixed with absolute ethyl alcohol for 20 min. The fixed cells in confocal dishes were washed 3 times with DPBS, then incubated in 0.2% Triton X-100 solution for 10 min. Next, the cells were washed three times and incubated in 5% bovine serum albumin (BSA) solution for 1 h, followed by incubating in antibody solution (USP11, 1:50 or p21, 1:100) for 8 h at 4 °C. Then the cells were washed three times and subsequently incubated in secondary antibody solution (DyLight 488, 1:1000 or DyLight 594, 1:1000) at room temperature for 2 h. Subsequently, the cells were incubated in Hoechst solution for 5 min followed by 3 washes with DPBS. Images were acquired with confocal microscope (NIKON).

### Cellular Fractionation

Cells were harvested from the 6 cm dishes, then were resuspended in 500 µL of buffer A containing the phosphatase inhibitor (Roche 4906845001) and protease inhibitors (Roche 4693159001). Buffer A was prepared according to the reference procedure 47. The reaction was mixed and incubated in an ice bath for 20 min. After that, the resultant mixture was vortexed (20 s) and centrifuged (15,000 rpm, 15 min). The supernatant (cytoplasmic fraction) was collected, and the precipitate was washed three times (5 min each time) with 400 µL buffer B. Then precipitate resuspended in 80 µL of buffer C containing the phosphatase inhibitors and protease inhibitors and vortexed for 30 min in an ice bath. Buffer B and C was prepared according to the reference methods 14. After centrifugation, the supernatant (nuclear fraction) was collected.

### Western blot analysis and Immunoprecipitation

The procedures of western blot analysis and immunoprecipitation were implemented according to previously reported 34, 41. Briefly, the cytoplasmic protein was prepared for immunoprecipitation with USP11 or p21 antibodies overnight at 4 °C. Next, the reactants mixed with protein G-magnetic beads at room temperature for 1 hour, followed by 3 washes with TBST, then the beads were boiled in 2× SDS loading buffer for 10 min and analyzed by western blotting with specific antibodies.

### Ubiquitination Assay

The procedure of ubiquitination assay was performed as previously described 34. Briefly, MCF-7 and BT474 cells were infected with negative control (NC) lentivirus or USP11-shRNA lentivirus for 48 h. After treatment with 20 μM MG132 for 6 h, cytoplasmic proteins were extracted by buffer A 47. p21 antibody was used to immunoprecipitate the cytoplasmic protein.

### Cycloheximide and MG132 treatment assays

Cells infected with indicated lentiviral shRNAs were seeded in 12-well plates (Nest Biotechnology, China). Later, the cells were cultured in medium containing 50 μg/mL CHX (Xiya Reagent Corporation, catalog no. 1014554) or 20 μM MG132 (Sigma catalog no. M8699) for the indicated times, followed by western blot analysis.

### Colony Formation Assay

MCF-7 cells were seeded in 12-well plates. After two weeks, the cell clones were fixed with absolute ethyl alcohol for 20 min. Next, the cell clones were stained with 0.2% crystal violet solution, and the number of colonies was counted using ImageJ.

### Xenograft models

All animal experiments were approved by the Experimental Animal Ethics Committee of Hunan University (no. 1107271911007500) and carried out following the National Guidelines for Animal Usage in Research (China). Female BALB/c mice and female nude mice were purchased from Hunan SJA Laboratory Animal Corporation. *Usp11*^-/-^ mice were generated by Cyagen Biosciences Corporation. To generate breast cancer xenografts in female mice, 1×10^7^ MCF-7 cells stably expressing the indicated shRNA or 1×10^6^ 4T1 cells were harvested and washed three times with DPBS. After suspending in serum-free media (100 μL), the cell suspension injected into the mice. WT mice were treated with DMSO or MTX (10 mg/kg, every 3 days); *Usp11* KO mice were treated with DMSO. Treatments were given by intraperitoneal injection. The tumor diameter was measured using a vernier caliper, and the tumor volume (*V*) was calculated according to the following formula: *V* = (*L* × *W*^2^)/2. The *L* represents the longest diameter and the *W* represents the shortest diameter.

### Immunohistochemistry

Breast cancer tissue microarray (HBreD090CS01) was purchased from Shanghai Outdo Biotech Company. Immunohistochemistry was performed using standard protocols 34. The USP11 and p21 expression were assessed by two pathologists. Images were analysed by ImageJ and IHC Profiler 48.

### Statistics

Quantitative variables were analyzed by Student's t-test between groups. One way analysis of variance (ANOVA) was used for comparisons each group. All of the data were processed by GraphPad Prism 8. A value of p < 0.05 was considered to be statistically significant. The significance level was presented as ^***^p < 0.001, ^**^p < 0.01 and ^*^p < 0.05.

## Results

### Correlation between p21 and USP11 in breast cancers

**Figure 1:**
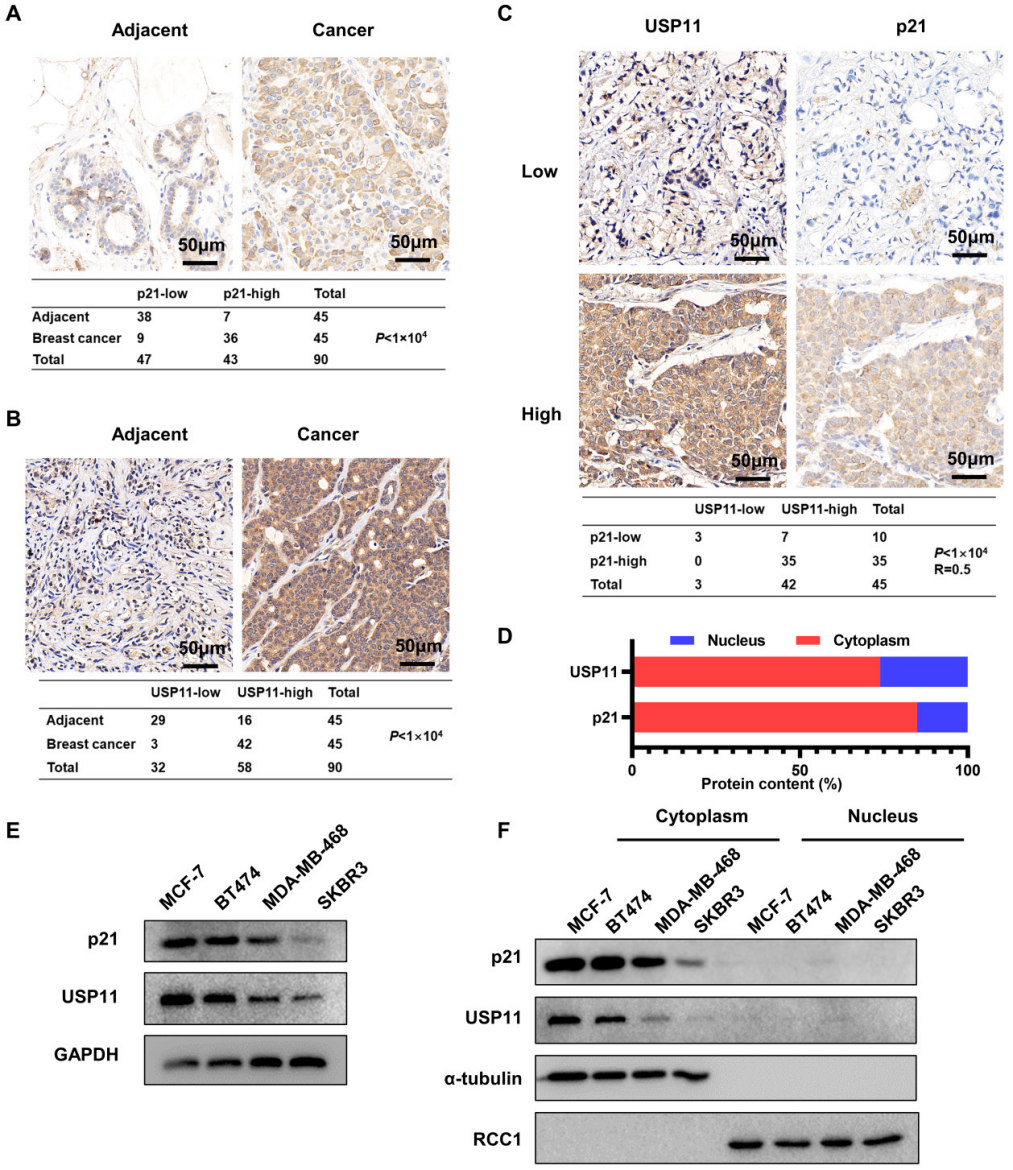
** p21 expression is correlated with USP11 in breast cancer. (A and B)** Representative immunohistochemical images of p21 (A) or USP11 (B) in breast cancer tissues and matched adjacent tissues. **(C)** Representative immunohistochemical images of p21 and USP11 in breast cancer tissues (n = 45). **(D)** The ratio of p21 and USP11 localization in the nucleus and cytoplasm of breast cancer tissues. **(E)** Expression of USP11 and p21 in breast cancer cell lines. **(F)** Expression of USP11 and p21 in cytoplasm and nucleus of breast cancer cell lines.

To investigate the potential physiological and pathological functions of p21 and USP11 in breast cancer, we performed bioinformatic analysis using the Clinical Proteomic Tumor Analysis Consortium (CPTAC) dataset. As shown in Figure S1A and S1B, both p21 and USP11 were significantly more highly expressed in clinical breast cancer tissues than in normal breast tissues. Meanwhile, the expression levels of p21 and USP11 were detected in 45 pairs of breast cancer tissues and adjacent normal tissues. Consistent with the above result, significantly higher p21 and USP11 levels were observed in breast cancer tissues than in adjacent normal tissues (Figure 1A-B). Moreover, increased p21 or USP11 expression was associated with higher clinical stages (Figure S1C), and there was a significant positive correlation between p21 and USP11 in breast cancer tissues (Figure 1C). In agreement with previous studies 26, 43, our data showed that both p21 and USP11 were mainly localized in the cytoplasm of breast cancer tissues (Figure 1D). Furthermore, we characterized p21 and USP11 expression in different breast cancer cell lines. Varying levels of p21 and USP11 expression were observed, with the highest expression occurring in MCF-7 cells, followed by BT474, MDA-MB-468 and SKBR3 cells. Interestingly, the expression levels of p21 were highly consistent with the expression of USP11 (Figure 1E), and both p21 and USP11 were mainly located in the cytoplasm of all breast cancer cell lines evaluated (Figure 1F). These data indicate that cytoplasmic p21 expression may be tightly correlated with USP11 expression in breast cancer.

### USP11 is phosphorylated by activated ERK1/2 at serine 905

**Figure 2:**
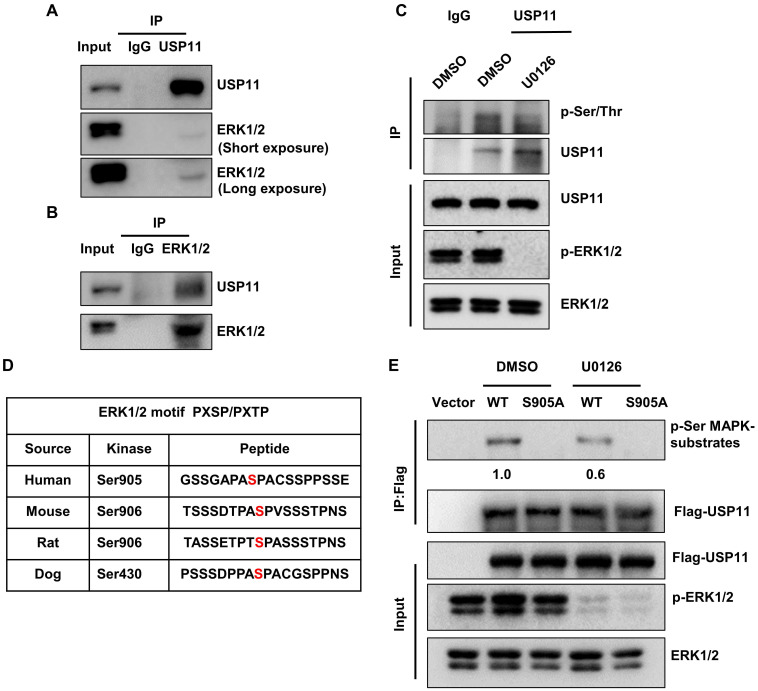
** USP11 is phosphorylated by activated ERK1/2 at serine 905. (A and B)** MCF-7 cell lysates were immunoprecipitated using anti-USP11 (A) or anti-ERK1/2 antibody (B). The immunoprecipitates were then examined with indicated antibodies. **(C)** MCF-7 cells were treated with DMSO or U0126 (5 µM) for 6 h. Cell lysates were immunoprecipitated with anti-USP11 antibody, followed by western blotting with anti-p-Ser/Thr antibody and other indicated antibodies. **(D)** Sequence alignment of the ERK1/2 phosphorylation site within USP11 orthologs from different species. **(E)** MCF-7 cells were transfected with Flag-USP11^wt^, Flag-USP11^S905A^, empty vector plasmids for 24 h, and then treated with DMSO or U0126 (5 µM) for 6 h. Cell lysates were immunoprecipitated using anti-Flag antibody, followed by western blotting with anti-phospho-MAPK/CDK substrates antibody and other indicated antibodies.

Previous studies have uncovered that the ERK signaling pathway plays key roles in the malignancy of breast cancer. To investigate the association between USP11 and ERK, a co-immunoprecipitation assay was carried out using an anti-USP11 antibody. Interestingly, ERK1/2 was present in the USP11 immunoprecipitates (Figure 2A). Reciprocal immunoprecipitation with ERK1/2 also brought down USP11 (Figure 2B). These results indicated that USP11 interacts with ERK1/2. Interestingly, when ERK1/2 phosphorylation was blocked with ERK inhibitor U0126 treatment, the phosphorylation levels of USP11 were downregulated (Figure 2C), suggesting that USP11 may be phosphorylated by ERK1/2.

To identify the putative sites of USP11 phosphorylated by ERK1/2, USP11 amino acid sequences were scanned by the Scansite databases (GPS 5.0) 49 for ERK1/2 consensus phosphorylation motifs. The results revealed that human USP11 contains a consensus phosphorylation site at serine 905 (Figure 2D), conforming to the optimal ERK1/2 motif PXSP/PXTP 50. To examine whether ERK1/2 actually induces USP11 phosphorylation, we introduced Flag-USP11^wt^ or Flag-USP11^S905A^ into MCF-7 cells and then carried out IP with an anti-Flag antibody. The phosphorylation levels of USP11 were determined using a phosphorylated motif antibody that recognized proteins containing the optimal ERK1/2 phosphorylation consensus motif. As expected, the phosphorylation levels of wild-type USP11 (USP11^wt^) were significantly decreased after U0126 treatment, and the USP11 mutant (USP11^S905A^) with a serine to aspartic acid at position 905 could not be phosphorylated by ERK1/2 (Figure 2E), demonstrating that serine 905 of USP11 is an ERK1/2 phosphorylation site. These data suggest that ERK1/2 interacts with USP11, which induces phosphorylation of USP11 at serine 905.

### USP11 phosphorylation by ERK1/2 redirects its subcellular localization

As mentioned above, USP11 was mainly localized in the cytoplasm of breast cancer cells, which is different from previous studies describing its nuclear location 34, 51-53. This prompted us to investigate how USP11 is retained in the cytoplasm. Given the strong positive correlation between p21 and USP11 in the cytoplasm, we therefore asked whether p21 affects the subcellular localization of USP11. However, neither knockdown of p21 nor overexpression of p21 with a NLS deletion mutation had any effect on USP11 localization (Figure S2A-B). Moreover, USP11 did not affect p21 localization after blocking the degradation of p21 with MG132 (Figure S2C-D).

**Figure 3:**
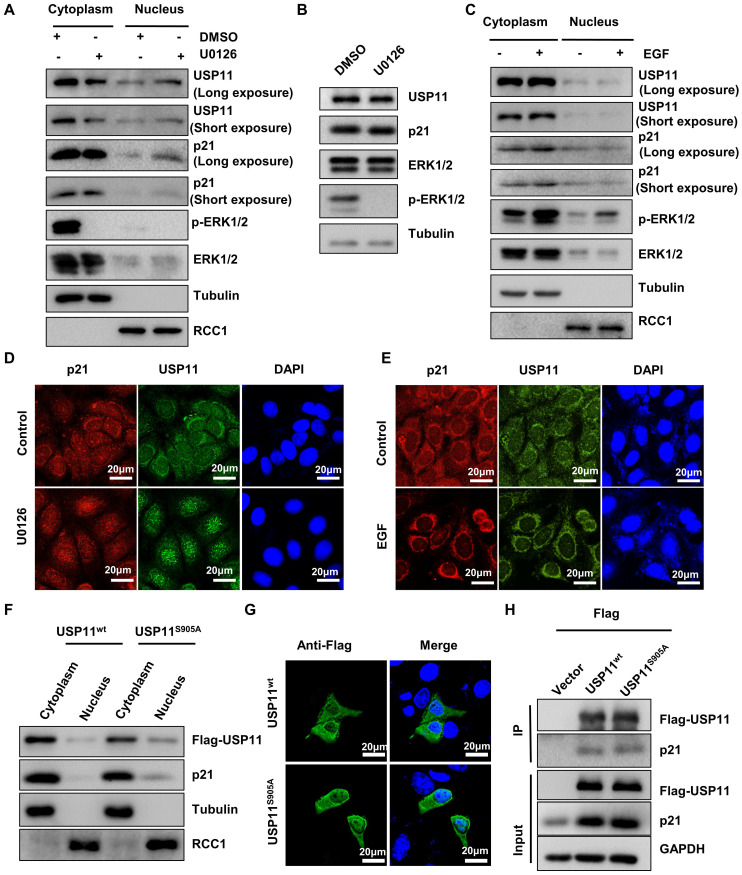
** USP11 phosphorylation by ERK1/2 redirects its subcellular localization. (A and B)** MCF-7 cells were treated with DMSO or U0126 (5 µM) for 6 h. The cytoplasmic and nuclear proteins (A) were analyzed by western blotting with indicated antibodies. Total proteins (B) were analyzed by western blotting with indicated antibodies. **(C)** MCF-7 cells were treated with EGF (10 ng/mL) or control for 30 min. The cytoplasmic and nuclear proteins were then analyzed by western blotting with indicated antibodies. **(D and E)** MCF-7 cells incubated with 5 µM U0126 for 6 h (D) or 10 ng/mL EGF for 30 min **(E)** were analyzed by immunofluorescent staining with anti-USP11 and anti-p21 antibodies. **(F, G and H)** Flag-USP11^WT^, Flag-USP11^S905A^ or empty vector plasmids were transfected into MCF-7 cells. The cytoplasmic and nuclear proteins were then analyzed by western blotting with indicated antibodies (F). The MCF-7 cells were then examined by immunofluorescence staining with anti-Flag antibody (G). Co-immunoprecipitation assays were carried out using anti-Flag antibody in MCF-7 cells lysates. The immunoprecipitates were then examined by western blotting (H).

To identify whether ERK1/2-phosphorylated USP11 affected its intracellular distribution, cytoplasmic and nuclear fractions were extracted from MCF-7 cells treated with U0126 or control. Strikingly, inhibition of USP11 phosphorylation mediated by U0126 caused a decrease of USP11 in the cytoplasm and an increase of USP11 in the nucleus, accompanied by consistent changes of p21 (Figure 3A). Of note, the changes in the subcellular distribution of USP11 and p21 were not derived from alterations in their protein levels because U0126 treatment had no effect on the expression levels of USP11 and p21 (Figure 3B). In contrast, ERK1/2 activated by EGF resulted in increased USP11 in the cytoplasm rather than in the nucleus (Figure 3C). The above results were further confirmed by immunofluorescent staining (Figure 3D-E).

To further verify that the subcellular location of USP11 is associated with its phosphorylation at the serine 905 site, MCF-7 cells were transfected with USP11^wt^ or USP11^S905A^. As shown in Figure 3F, USP11^S905A^ was reduced in the cytoplasm and increased in the nucleus compared with USP11^WT^, which was consistent with immunofluorescent staining results indicating the subcellular location of USP11^wt^ and USP11^S905A^ (Figure 3G). However, phosphorylation of USP11 at the serine 905 site did not affect the interaction between USP11 and p21 (Figure 3H), implying that phosphorylation of USP11 by ERK1/2 did not enhance its effect on p21. Taken together, phosphorylation of USP11 by ERK1/2 is essential to promote its cytoplasmic localization.

### Cytoplasmic p21 interacts with USP11 in breast cancer cells

**Figure 4:**
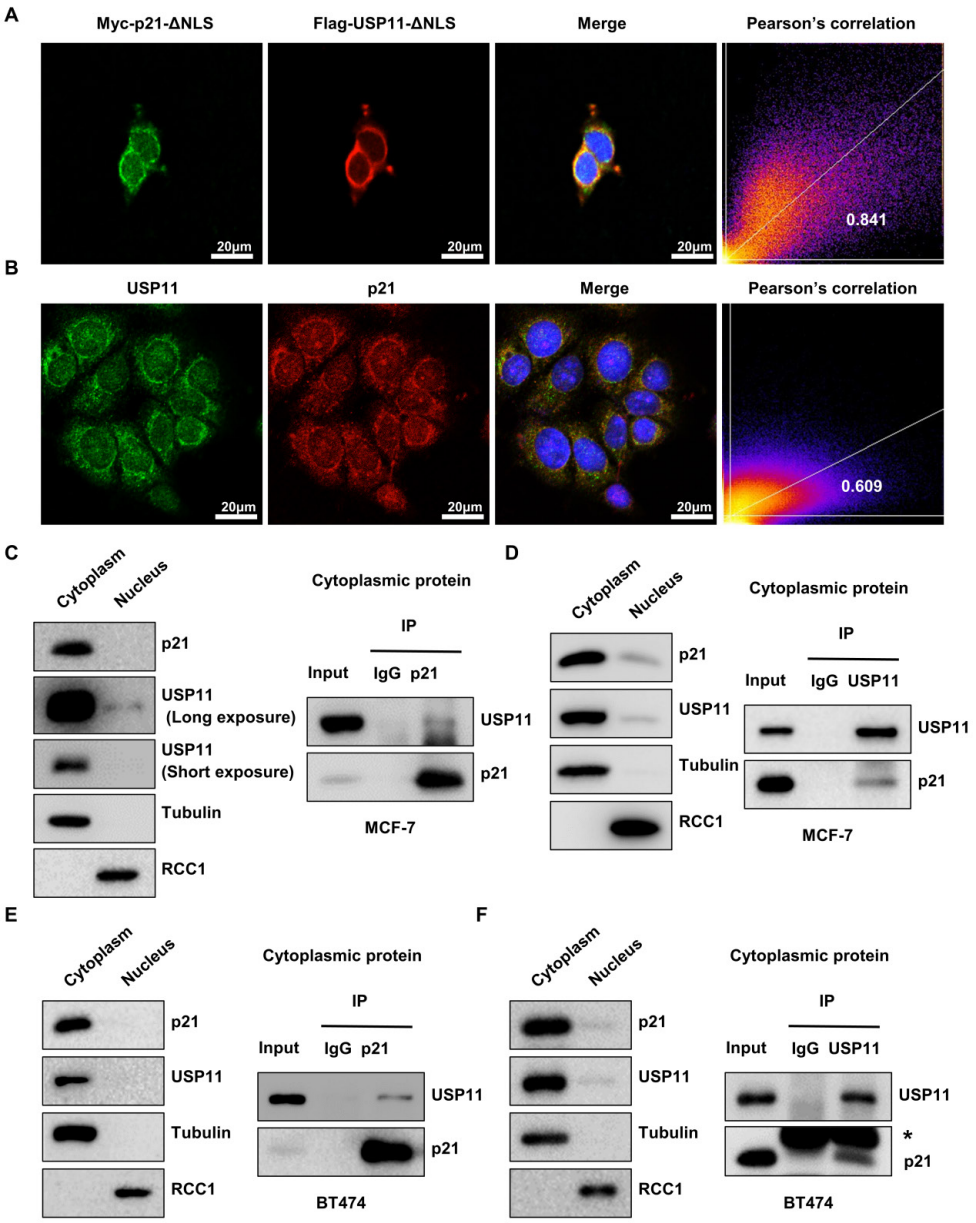
** Cytoplasmic p21 interacts with USP11. (A)** Myc-p21-ΔNLS or Flag-USP11-ΔNLS plasmids were transfected into MCF-7 cells. The MCF-7 cells were then examined by immunofluorescence staining with anti-Flag and anti-Myc antibodies. **(B)** The MCF-7 cells were examined by immunofluorescence staining with anti-USP11 and anti-p21 antibodies. **(C and D)** Co-immunoprecipitation assays were carried out using anti-p21 (C) or anti-USP11 (D) antibody in cytoplasmic proteins of MCF-7 cells. The immunoprecipitates were then examined by western blotting. **(E and F)** Co-immunoprecipitation assays were carried out using anti-p21 (E) or anti-USP11 (F) antibody in cytoplasmic proteins of BT474 cells. The immunoprecipitates were then examined by western blotting. The “*” indicates antibody light chain.

Given the correlation between cytoplasmic p21 and USP11 in breast cancer tissues and cells, we hypothesized that cytoplasmic p21 might interact with USP11. To address this, we first examined the subcellular localization of p21 and USP11. MCF-7 cells were cotransfected with nuclear localization signal deletion mutants of USP11 (Flag-USP11-ΔNLS) and p21 (Myc-p21-ΔNLS). Immunofluorescent staining images indicated that p21-ΔNLS and USP11-ΔNLS were colocalized in the cytoplasm (Figure 4A). Furthermore, we found that the colocalization of endogenous p21 and USP11 was also presented in the cytoplasm (Figure 4B).

To confirm that cytoplasmic p21 indeed interacts with USP11, Flag-USP11-ΔNLS or Myc-p21-ΔNLS plasmids were transfected into MCF-7 cells, and coimmunoprecipitation (co-IP) was carried out. As shown in Figure S3A, USP11 coimmunoprecipitated with ectopically expressed Myc-p21-ΔNLS in the cytoplasm. Reciprocal immunoprecipitation with exogenous Flag-USP11-ΔNLS in the cytoplasm also brought down p21 (Figure S3B). To test the association of endogenous p21 and USP11, cytoplasmic protein was extracted from MCF-7 and BT474 cells for co-IP. As expected, USP11 was detected in the anti-p21 immunoprecipitates and vice versa, rather than in an isotype-matched negative control IgG (Figure 4C-F). Taken together, these data indicate that USP11 interacts with p21 in the cytoplasm of breast cancer cells.

### USP11 affects the protein levels of cytoplasmic p21

**Figure 5:**
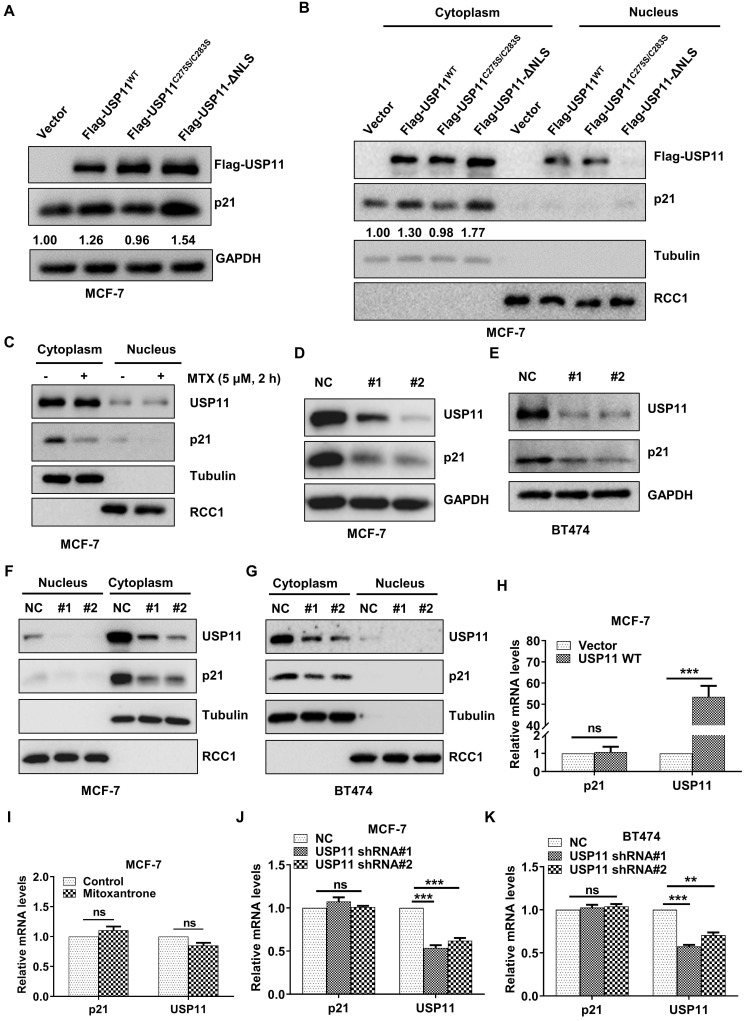
** USP11 regulates the protein levels of cytoplasmic p21 in breast cancer. (A and B)** Flag-USP11^WT^, Flag-USP11^C275S/C283S^, Flag-USP11-ΔNLS or empty vector plasmids were transfected into MCF-7 cells, respectively. Total proteins (A) or cytoplasmic/nuclear proteins (B) were extracted and subjected to western blotting using the indicated antibodies. **(C)** MCF-7 cells were incubated with 5 µM mitoxantrone for 2 h. Cytoplasmic and nuclear proteins were then examined by western blotting using indicated antibodies. **(D and E)** Total protein from MCF-7 (D) or BT474 (E) cells with or without USP11 knockdown were extracted and analyzed by western blotting using indicated antibodies. **(F and G)** MCF-7 (F) and BT474 (G) cells were infected with lentiviral shRNAs against USP11 or control. Cytoplasmic and nuclear proteins were extracted and analyzed by western blotting using indicated antibodies. **(H)** mRNA levels of p21 and USP11 in MCF-7 cells with or without overexpressing USP11^WT^ were analyzed by qRT-PCR. **(I)** mRNA levels of p21 and USP11 in MCF-7 cells treated with 5 µM mitoxantrone or control for 2 h were analyzed by qRT-PCR. **(J and K)** mRNA levels of p21 and USP11 in MCF-7 (J) and BT474 (K) cells with or without USP11 knockdown were analyzed by qRT-PCR. Values for samples are presented as mean ± SD, ns, no significant difference, one-way analysis of variance (ANOVA), **p < 0.01, ***p < 0.001.

Protein-protein interactions are important for regulating p21 levels 54, 55. Based on the interaction between USP11 and p21 in the cytoplasm identified above, we next investigated the effect of USP11 on cytoplasmic p21 levels. Wild-type and mutant USP11 were introduced into MCF-7 cells. As expected, overexpression of USP11 and USP11-ΔNLS led to an increase in total p21 levels (Figure 5A) and cytoplasmic p21 levels (Figure 5B). In contrast, overexpression of a catalytically inactive USP11^C275S/C283S^ showed no effects on total p21 levels or cytoplasmic p21 levels (Figure 5A-B). Notably, USP11-ΔNLS caused a stronger increase in cytoplasmic p21 levels, which might be derived from the fact that USP11-ΔNLS was mainly expressed in the cytoplasm (Figure 5B). Furthermore, we treated MCF-7 cells with mitoxantrone, an inhibitor of the deubiquitinating activity of USP11, and cytoplasmic p21 markedly decreased after mitoxantrone exposure (Figure 5C).

To further clarify the role of USP11 in regulating cytoplasmic p21 levels, endogenous USP11 was knocked down using two short hairpin RNAs (shRNAs). As shown in Figure 5D and 5E, USP11 downregulation significantly diminished the total p21 levels. Subcellular fraction assays demonstrated that the effect of USP11 ablation on p21 occurred mainly in the cytoplasm (Figure 5F-G). Meanwhile, USP11 overexpression, USP11 inhibition or USP11 knockdown did not affect the p21 mRNA levels (Figure 5H-K), which indicated that USP11 regulated p21 expression at the posttranslational level rather than at the transcriptional level. These findings indicate that USP11 remarkably affects the levels of cytoplasmic p21 in breast cancer cells, which is dependent on its enzymatic activity.

### USP11 stabilizes cytoplasmic p21 through deubiquitination

**Figure 6:**
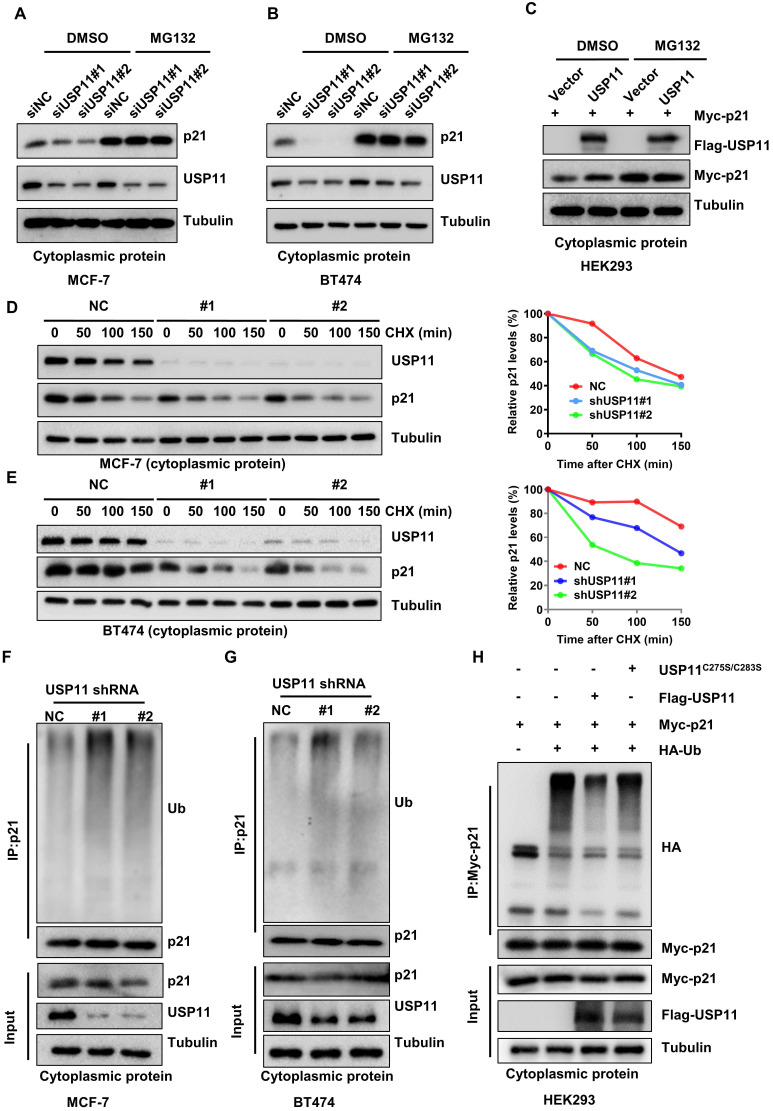
** USP11 stabilizes cytoplasmic p21 by deubiquitination. (A and B)** MCF-7 (A) and BT474 (B) cells with or without USP11 knockdown were treated with DMSO or 20 µM MG132 for 6 h. Cytoplasmic protein was extracted and subjected to western blotting analysis using indicated antibodies. **(C)** HEK293 cells transfected with a Flag-USP11 or empty vector plasmids for 24 h were treated with DMSO or MG132 for 6 h. Cytoplasm proteins were extracted and subjected to western blotting analysis using indicated antibodies. **(D and E)** MCF-7 (D) and BT474 (E) cells with or without USP11 knockdown were incubated with CHX (50 µg/mL). Cytoplasm proteins were extracted at the indicated time points and analyzed by western blotting using indicated antibodies. Right: relative p21 levels in the cytoplasm (normalized to tubulin) were determined. **(F and G)** MCF-7 (F) and BT474 (G) cells with or without USP11 knockdown were treated with MG132 (20 µM) for 6 h. Cytoplasmic protein was extracted and subjected to immunoprecipitation with anti-p21 antibody. The immunoprecipitates were then examined by western blotting using the indicated antibodies. **(H)** HEK293 cells transfected with Myc-p21, Flag-USP11^WT^, Flag-USP11^C275S/C283S^ and HA-Ub plasmids were treated with MG132 (20 µM) for 6 h. Cytoplasmic protein was extracted and subjected to immunoprecipitation with anti-HA antibody. The immunoprecipitates were then examined by western blotting using indicated antibodies.

To validate whether USP11 affects cytoplasmic p21 expression in a proteasome-dependent manner, MCF-7 and BT474 cells with or without USP11 were treated with the proteasome inhibitor MG132 to block protein degradation. As expected, downregulation of total p21 or cytoplasmic p21 levels caused by USP11 knockdown (Figure S4A-B; Figure 6A-B) and upregulation of cytoplasmic p21 caused by USP11 overexpression (Figure 6C) could be abolished by MG132, suggesting that USP11 affected cytoplasmic p21 expression by regulating its proteasomal degradation. Subsequently, we treated the indicated cells with the protein synthesis inhibitor cycloheximide (CHX). USP11 knockdown significantly decreased the half-life of cytoplasmic p21 in MCF-7 and BT474 cells (Figure 6D-E). Furthermore, the levels of cytoplasmic p21 polyubiquitylation were measured. Knockdown of USP11 caused an apparent increase in the polyubiquitination of cytoplasmic p21 in MCF-7 and BT474 cells (Figure 6F-G). Conversely, the overexpression of USP11 significantly diminished the polyubiquitinated levels of cytoplasmic p21 (Figure 6H). Of note, USP11^C275S/C283S^ failed to prevent cytoplasmic p21 ubiquitination (Figure 6H), suggesting that the enzymatic activity of USP11 is critical for the deubiquitination of cytoplasmic p21. Collectively, these findings indicate that USP11 promotes cytoplasmic p21 stabilization by deubiquitination in breast cancer cells.

### USP11 accelerates breast cancer cells growth by regulating cytoplasmic p21 *in vivo*

**Figure 7:**
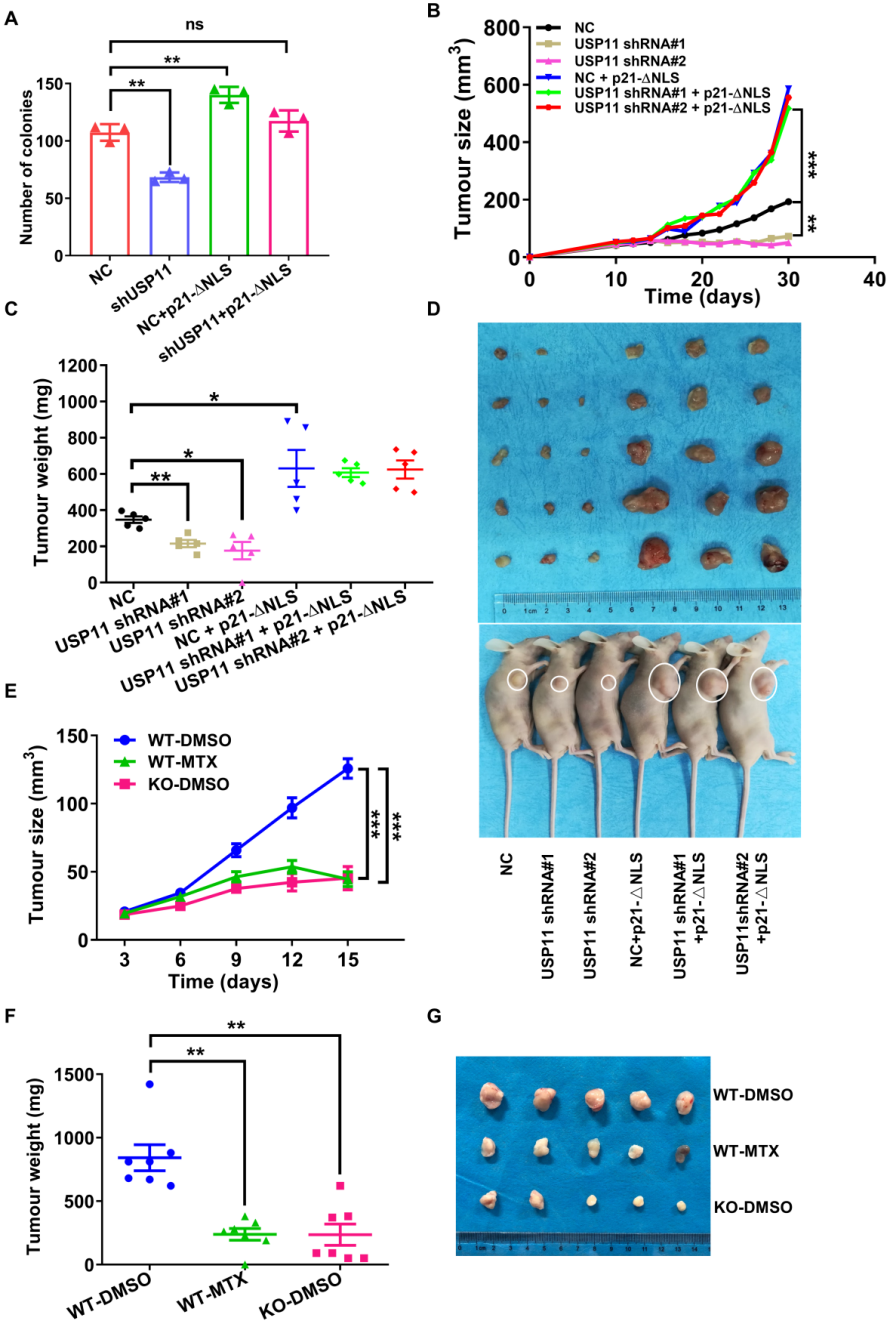
** USP11 promotes breast cancer cell proliferation by regulating cytoplasmic p21. (A)** The proliferation of MCF-7 cells infected with shRNA or/and p21-ΔNLS expressing lentiviral constructs were determined by colony formation assay. **(B-D)** Growth (B), weight (C), and images (D) of xenograft tumors formed by MCF-7 cells infected with shRNA or/and p21-ΔNLS expressing lentiviral constructs were shown. **(E-G)** The 1×10^6^ 4T1 cells were orthotopically injected into mice. WT mice were treated with DMSO or mitoxantrone (MTX), and *Usp11* KO mice were treated with DMSO. Growth (E), weight (F), and images (G) of xenograft tumors were show. Values for representative samples are presented as mean ± SD, ns: no significant difference, one-way analysis of variance (ANOVA), *p < 0.05, **p < 0.01 and ***p < 0.001.

Cytoplasmic p21 was reported to act as an oncogenic protein in various cancers 19, 22, 56. Since USP11 regulates cytoplasmic p21 stability, we hypothesized that USP11 may promote breast cancer cell proliferation through cytoplasmic p21. To examine this hypothesis, we conducted a colony formation assay. The results presented that USP11 depletion severely suppressed MCF-7 cells proliferation, and that p21-ΔNLS reintroduction could reversed the effect induced by USP11 depletion (Figure 7A). Conversely, USP11 overexpression enhanced the proliferation of MCF-7 cells but not p21-depleted cells (Figure S5A). To demonstrate the function of USP11 *in vivo*, USP11-depleted and/or p21-ΔNLS-overexpressing MCF-7 cells (Figure S5B) were subcutaneously injected into nude mice, and tumor growth was closely monitored at the indicated time points. Compared with mice inoculated with control cells, mice bearing USP11-depeleted MCF-7 cells showed decreased tumor growth throughout the experiment (Figure 7B). Excised xenograft tumors were measured 30 days after tumor cell implantation, the size and weight of the tumor formed by USP11-depleted MCF-7 cells were significantly decreased (Figure 7C-D). Of note, reintroducing p21-ΔNLS into USP11-depeleted cells reversed the tumor-inhibiting effect of USP11 depletion (Figure 7B-D).

The tumor microenvironment is important for tumor progression 57. To assess whether USP11 could influence tumorigenesis within the tumor microenvironment, the mouse breast cancer cell line 4T1 was transplanted into *Usp11*-WT or *Usp11*-KO mice. The results showed that USP11 deficiency suppressed the growth of 4T1 cells *in vivo* (Figure 7E-G). These data suggested that USP11 not only played an oncogenic role in breast cancer cells, but also promoted the development of breast cancer by affecting the growth environment of breast cancer cells.

To further test the feasibility of developing USP11 inhibitor for breast cancer therapy, we used the USP11 inhibitor MTX to treat mice for two weeks. The results showed that USP11 inhibition effectively attenuated the volume and weight of the tumors formed by 4T1 cells (Figure 7E-G). To further investigate the abundance of USP11 and p21 in the xenograft tumor tissues, immunohistochemical staining was performed. As expected, USP11 inhibition caused a decrease of p21 in the cytoplasm and consequent proliferation inhibition, as indicated by Ki67 staining (Figure S5C). Taken together, these data demonstrate that USP11 promotes breast cancer cell growth *in vivo* through cytoplasmic p21, and USP11 inhibitor MTX has promise for potential applications in the treatment of breast cancer.

## Discussion

The ERK1/2 pathway plays central roles in cell proliferation. A previous study showed that overexpression and hyperphosphorylation of ERK1/2 is frequently found in breast cancer 58. ERK2 phosphorylates p21 at the Thr57 and Ser130 sites to promote its cytoplasmic localization 30. Intriguingly, our results showed that USP11 could be phosphorylated by ERK1/2 at the Ser905 site and led to its cytoplasmic retention. This is critical for stabilizing cytoplasmic p21 levels because inhibition of USP11 phosphorylation mediated by ERK1/2 not only caused a decrease of USP11 in the cytoplasm but also resulted in a downregulation of cytoplasmic p21 levels. Of note, the cytoplasmic retention of USP11 did not depend on p21, and vice versa. Thus, the subcellular localization between USP11 and p21 seems to be independent of each other. In addition, since inhibition of ERK1/2 could not completely abolish the cytoplasmic localization of USP11, we are unable to exclude the possibility that other mechanisms are involved in regulating the subcellular distribution of USP11.

p21 is a labile protein with a half-life of about 30 min 59 that is degraded mainly via the ubiquitin-proteasome pathway 60. In the nucleus, three E3 ligases complexes, SCK^SKP2^, APC/C^CDC20^ and CRL4^CDT2^, are involved in p21 ubiquitination and degradation. Our previous study further demonstrated that USP11 could block p21 degradation mediated by SCK^SKP2^, APC/C^CDC20^ and CRL4^CDT2^ by removing the polyubiquitin chains bound to p21, and thus stabilize p21 in the nucleus, revealing that p21 levels in the nucleus are regulated in a dynamic balanced manner 34.

Unlike the three identified E3 ligases in the nucleus, only one E3 ubiquitin ligase, CRL2^LRR1^, has been reported to specifically target cytoplasmic p21 for its ubiquitination and subsequent degradation. In this study, we found that USP11 was mainly located in the cytoplasm of breast cancer cells. Similar to its function in the nucleus, USP11 interacted with and stabilized cytoplasmic p21 by removing p21 polyubiquitination chains. To our knowledge, these results are the first evidence that ubiquitinated p21 in the cytoplasm can be recycled through deubiquitination. It would be interesting to further test whether USP11 protects cytoplasmic p21 from the degradation mediated by CRL2^LRR1^, which will contribute to elucidating the dynamic regulatory mechanism of cytoplasmic p21 levels.

It has been reported that USP11 functions as a tumor suppressor and an oncogenic protein. The tumor-suppressive activities of USP11 were found in non-small cell lung cancer 34, squamous cell carcinoma 39 and brain tumors 38. In contrast, USP11 plays a tumor-promoting role in hepatocellular carcinoma 40, melanoma 41, gastric cancer 42 and breast cancer 43. Thus, USP11 exhibits two seemingly contradictory effects on tumor development. Interestingly, its biological function is similar to the dual behavior of p21. Given the intracellular interactions and consistent subcellular distributions between USP11 and p21, we speculate that USP11's role as a tumor suppressor is associated with its nuclear localization and nuclear p21, such as in lung cancer, whereas the cytoplasmic localization of USP11 contributes to oncogenic effects through cytoplasmic p21, such as in breast cancer. However, further investigations in additional types of tumors are required to clarify the relationship between subcellular distribution of USP11 and its biological functions.

In summary, we provide the first evidence that ubiquitinated p21 in the cytoplasm can be recycled through USP11-mediated deubiquitination. Based on our current and previous results 34, we propose a model to clarify the different biological functions of USP11 by regulating cytoplasmic and nuclear p21 levels. The activated ERK1/2 phosphorylated USP11 at Ser905 site, which promoted the cytoplasmic localization of USP11. As a result, USP11 enhances tumor cell proliferation by deubiquitinating and stabilizing cytoplasmic p21. Conversely, inhibition of USP11 phosphorylation mediated by ERK1/2 contributes to its nuclear localization. In the nucleus, USP11 causes the stabilization of nuclear p21 by reversing p21 polyubiquitination and acts as tumor suppressor by regulating cell cycle progression.

## Supplementary Material

Supplementary figures and tables.

## Abbreviations

CPTAC: clinical Proteomic Tumor Analysis Consortium
qRT-PCR: Quantitative Real‑time PCR
NC: negative control
CHX: cycloheximide
MTX: mitoxantrone
co-IP: coimmunoprecipitation
shRNA: specific short hairpin RNA
KO: knockout
WT: wild-type
