# Supplementary material for: ERK-mediated Cytoplasmic Retention of USP11 Contributes to Breast Cancer Cell Proliferation by Stabilizing Cytoplasmic p21
Source: Int J Biol Sci. 2022 Mar 21;18(6):2568–82. doi: 10.7150/ijbs.71327 (PMC8990481; doi:10.7150/ijbs.71327)
Supplement: Supplementary file 1 — Supplementary figures and tables. [file ijbsv18p2568s1.pdf]

Supplementrary materials

Supplementary figures

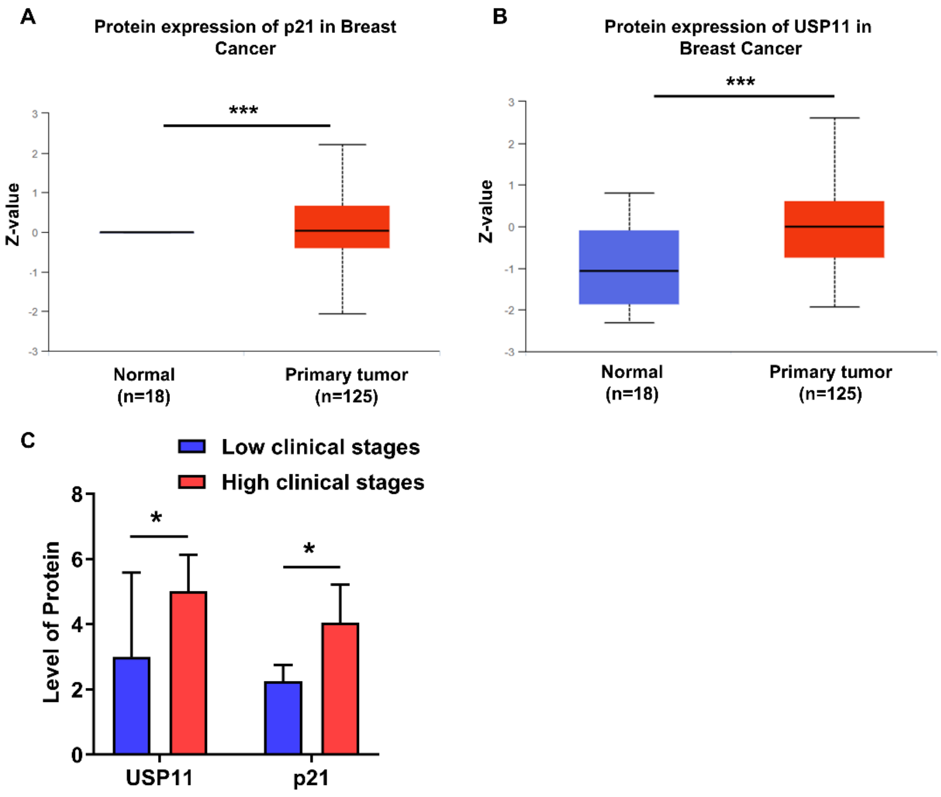

Figure S1 The clinical relevance of p21 and USP11 in breast cancer. (A and B) p21 (A) and USP11 (B) protein expression in breast cancer tissues were analyzed by CPTAC data from the ULCAN cancer database. (C) Expression levels of p21 and USP11 in breast cancer tissues of different clinical stages were shown. Low clinical stages refer to stage 1A and high clinical stages refer to stage 2A, 2B, 3A, and 3C. $*p < 0.05$, $***p < 0.001$.

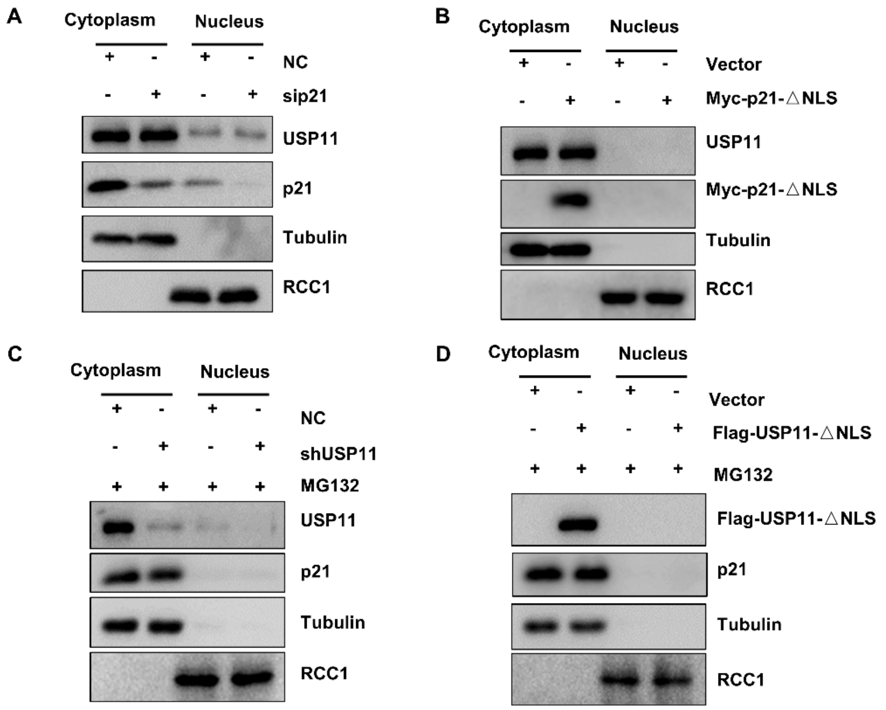

Figure S2 p21 and USP11 do not regulate each other's localization. (A) Cytoplasmic and nuclear proteins from MCF-7 cells with or without p21 knockdown were analyzed by western blotting with indicated antibodies. (B) Myc-p21-ΔNLS or empty vector plasmids were transfected into MCF-7 cells. Cytoplasmic and nuclear proteins were then analyzed by western blotting with indicated antibodies. (C) MCF-7 cells with or without USP11 knockdown were treated with DMSO or 20 μM MG132 for 6 h. Cytoplasmic and nuclear proteins were then analyzed by western blotting with indicated antibodies. (D) MCF-7 cells transfected with Flag-USP11-ΔNLS or empty vector plasmids were treated with DMSO or 20 μM MG132 for 6 h. Cytoplasmic and nuclear proteins were then analyzed by western blotting with indicated antibodies.

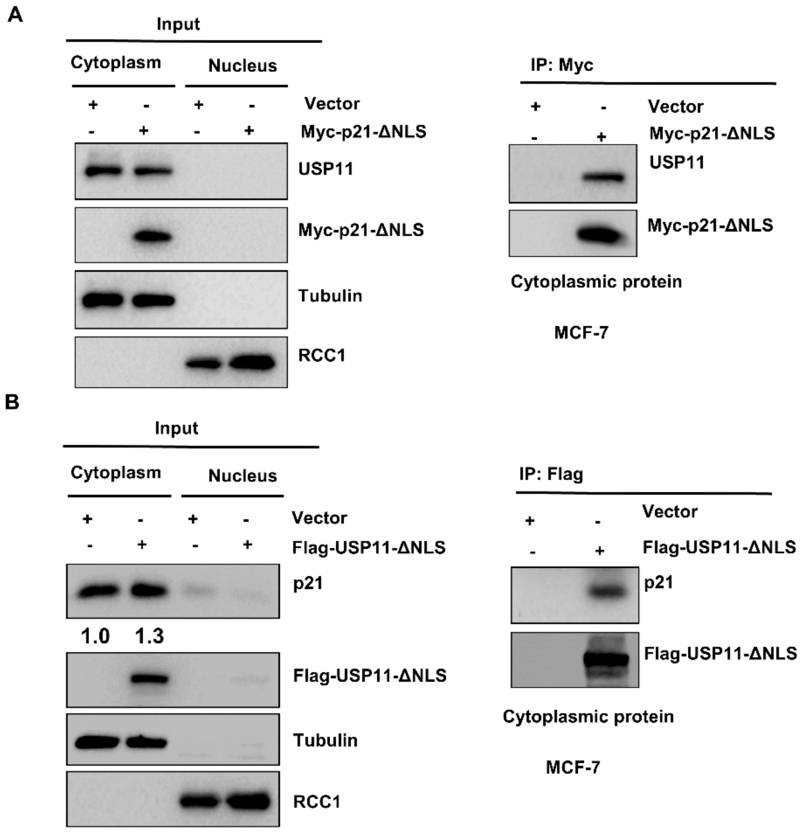

Figure S3 p21 interacts with USP11 in breast cancer cells. (A and B) MCF-7 cells were transfected with empty vector, Myc-p21-ΔNLS or Flag-USP11-ΔNLS plasmids. Cytoplasmic proteins were extracted and subjected to immunoprecipitation with anti-Myc (A) or anti-Flag (B) antibody.

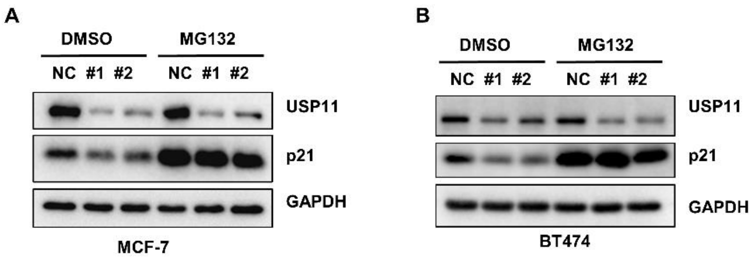

Figure S4 USP11 protects p21 from proteasome-dependent degradation in breast cancer cells. (A and B) Cells with or without USP11 knockdown were treated with MG132 (20 μM) for 6 h. Total protein was extracted from MCF-7 cells (A) or BT474 cells (B) and subjected to western blot analysis with indicated antibodies.

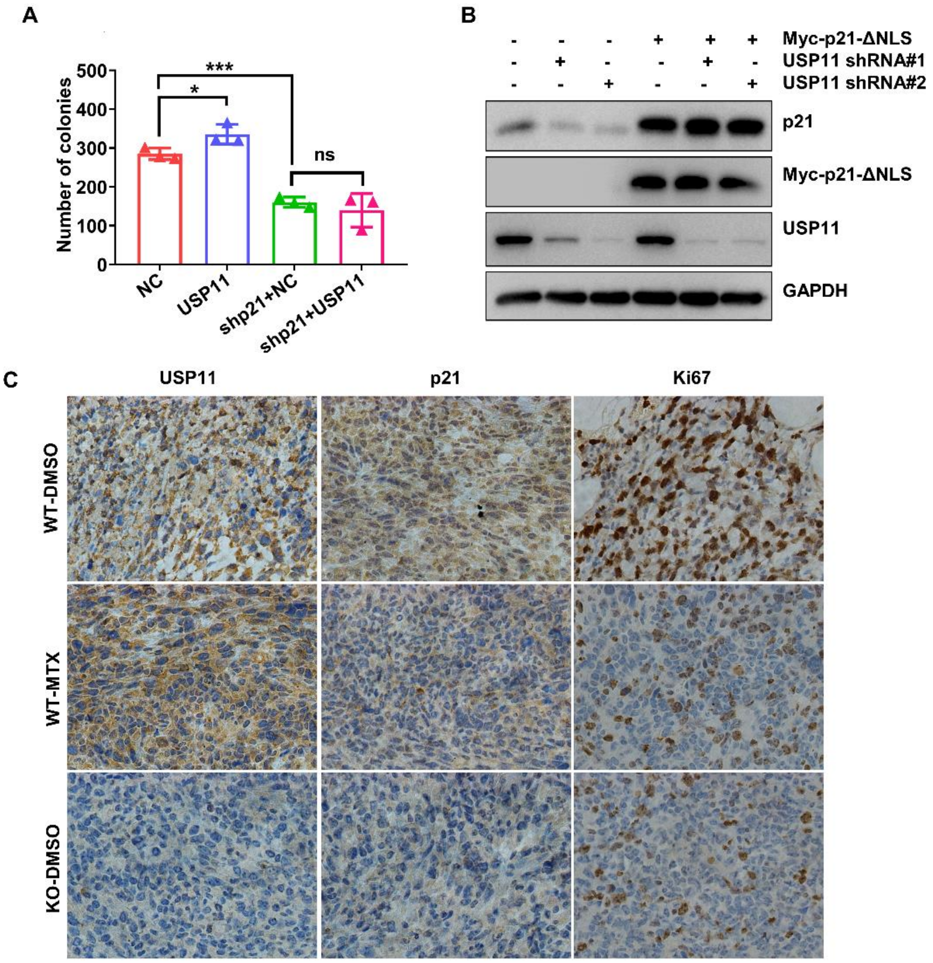

Figure S5 USP11 promotes breast cancer cell proliferation by regulating cytoplasmic p21. (A) Effects of USP11 and p21 on MCF-7 cell proliferation were analyzed by colony formation. (B) MCF-7 cells implanted into nude mice were lysed and analyzed by western blotting with indicated antibodies. (C) The protein levels of USP11, p21 and Ki67 in xenograft tumors formed by 4T1 cells were analyzed by immunohistochemistry. Values shown for representative samples are presented as mean ± SD, ns: no significant difference, one-way analysis of variance (ANOVA), $*p < 0.05$, $***p < 0.001$.

Supplementary tables

Table S1. siRNA target sequences.

Name	Sequences (5'-3')
**Negative control siRNA**	TTCTCCGAACGTGTCACGTTTC
**siUSP11#1**	AATGAGAATCAGATCGAGTCC
**siUSP11#2**	AAGGCAGCCTATGTCCTCTTC
**sip21**	CUUCGACUUUGUCACCGAG

Table S2. shRNA target sequences.

Name	Sequences (5'-3')
**Negative control shRNA**	TTCTCCGAACGTGTCACGT
**shUSP11#1**	CCGTGATGATATCTTCGTCTA
**shUSP11#2**	AAGGCAGCCTATGTCCTCTTC
**shp21**	CTTCGACTTTGTCACCGAG

Table S3. The primer sequences for quantitative real-time PCR.

Name	Sequences (5'-3')
**USP11-F**	AGGTGTCAGGTCGCATTGAG
**USP11-R**	TGAGAGCCGGTACATCAGGA
**GAPDH-F**	AAGGTGAAGGTCGGAGTCAA
**GAPDH-R**	AATGAAGGGGTCATTGATGG
**p21-F**	ATTAGCAGCGGAACAAGGAGTCAGACAT
**p21-R**	CTGTGAAAGACACAGAACAGTACAGGGT
